# The Role of Natural Products in the Improvement of Cancer-Associated Cachexia

**DOI:** 10.3390/ijms24108772

**Published:** 2023-05-15

**Authors:** Yohan Han, Hyo In Kim, Jinbong Park

**Affiliations:** 1 Department of Pharmacology, College of Korean Medicine, Kyung Hee University, Seoul 02447, Republic of Korea; 2 Department of Surgery, Beth Israel Deaconess Medical Center, Harvard Medical School, Boston, MA 02215, USA

**Keywords:** natural product, cancer-induced cachexia, muscle atrophy, adipose tissue atrophy, anticancer drug-induced cachexia, AMPK

## Abstract

The enormous library of natural products and herbal medicine prescriptions presents endless research avenues. However, the lack of research evidence and trials on cancer-induced cachexia limit the therapeutic potential of natural products. Cancer-induced cachexia is a systemic wasting syndrome characterized by continuous body weight loss with skeletal muscle and adipose tissue atrophy. Cancer cachexia is a problem in itself and reduces the quality of life by lessening the treatment efficacy of anticancer drugs. This review summarizes single natural product extracts for cancer-induced cachexia, not compounds derived from natural products and herbal medicine prescriptions. This article also discusses the effect of natural products on cachexia induced by anticancer drugs and the role of AMPK in cancer-induced cachexia. The article included the mice model used in each experiment to encourage researchers to utilize animal models for research on cancer-induced cachexia in the future.

## 1. Introduction

Cachexia is a wasting syndrome characterized by uncontrolled body weight loss in disease status. Clinically, cachexia is defined as when more than 10% of body weight is decreased due to a pathological reason [1]. The loss of homeostasis on energy balance is one of the most important factors. Cachexia is often accompanied by reduced food intake, decreased appetite, abnormal metabolic changes, excessive energy expenditure, increased catabolism, and inflammation. Such an alteration in whole-body metabolism differs from simple starvation and nutritional imbalance, which are easily handled by supplementing adequate nutrients [2]. Cachexia occurs in not only malignant diseases but also various chronic, non-malignant diseases, especially in advanced stages. For chronic diseases, such as heart failure, kidney disease, rheumatoid arthritis, chronic obstructive pulmonary disease (COPD), and AIDS, the incidence of cachexia ranges from 5 to 15%. More importantly, 15 to 40% of these patients die due to cachexia [3].

Regardless of the type, cancer also induces cachexia, which can seriously impact the patient’s quality of life. Prevalence ranges of advanced cancer from 50 to 80% and 20 to 80% mortality are reported in cancer patients who suffer from cachexia [4]. The worst aspect is that anticancer drugs can be a direct cause of cachexia, causing a significant reduction of its therapeutic effect as well as shortening the survival period [5]. Numerous studies have attempted to understand the pathological pathway of cancer-induced cachexia. Of note, the molecular mechanisms responsible for the occurrence of cancer-induced cachexia and its associated symptoms remain partially unknown [6]. Although not fully elucidated yet, inflammatory mediators such as cytokines are considered to play a major role in cachexia [7]. Increased serum levels of interleukin-6 (IL-6) or C-reactive protein (CRP) are observed in cancer-induced cachexia patients, and these cytokines are even used as diagnostic markers for cancer-induced cachexia [8]. Thus, approaches have been made to treat cancer-induced cachexia by targeting inflammation. Celecoxib, a nonsteroidal anti-inflammatory drug (NSAID) and a selective cyclooxygenase-2 (COX-2) inhibitor, was shown to exert beneficial actions in cancer-induced cachexia patients [9].

The most distinct pathological feature shown in cachexia is weight loss derived from the depletion of muscle and fat tissues [10]. The loss of skeletal muscle mass markedly reduces the survival period in addition to its normal function on movement. Moreover, the depletion of skeletal muscle negatively affects the response to therapies [11]. To maintain skeletal muscle in normal physiology, a tight homeostasis between protein degradation and synthesis is required [12]. In cachexia patients, the degradation of skeletal muscle proteins is driven more rapidly than synthesis. Protein degradation is mainly regulated by the ubiquitin/proteasome system (UPS) [13]. This UPS mechanism is caused by the sequential binding of ubiquitin with a ubiquitin-activating enzyme (E1), ubiquitin-conjugating enzyme (E2), and ubiquitin ligase (E3). E3 specifically recognizes the substrate protein and allows ubiquitin to be delivered to the target protein, thus causing the degradation of the skeletal muscle [14]. Two muscle-specific E3 are significantly elevated and serve as markers of muscle atrophy. One is muscle atrophy F-box (MAFbx/atrogin-1) and the other is muscle RING finger-1 (MuRF-1) [15]. MAFbx and MuRF1 in skeletal muscle induce degradation via 26S proteasome by binding to selective substrates for ubiquitination. Thus, the increased expression level of MAFbx and MuRF1 has been considered responsible for muscle loss [16]. Concomitantly, the anabolic insulin-like growth factor-1 (IGF-1) is decreased in both cancer patients and mouse models [17]. Similar to skeletal muscles, cardiac muscle is also degraded through the same UPS mechanism upon cardiac dysfunctions, such as heart failure and arrhythmia [18].

Atrophy of adipose tissue is also accompanied by cachexia. Cachexia induces abnormal lipid metabolism between lipid uptake and decreased fatty acid (FA) utilization, which then leads to lipid depletion. In patients with cachexia, high levels of blood glycerol, free FA, and triacylglycerol are observed [19]. In some sorts of cachexia cases, adipose tissue is more significantly decreased than that of muscle, and its remaining amount affects the survival period [20]. Humans have two types of adipose tissues: white adipose tissue (WAT), which stores energy, and brown adipose tissue (BAT), which consumes energy. ‘Browning’ is a phenomenon in which WAT exerts the characteristics of BAT upon specific physiological and pathological conditions. Pathological browning is altered in cachectic WAT, and lipid is consumed through uncoupling protein 1 (UCP1)-dependent non-shivering heat generation [21].

There is no standard treatment protocol for cancer-induced cachexia established yet. The ASCO guideline reviewed 20 systemic review articles and 13 randomized controlled trials to suggest a multi-modal approach of nutritional support (omega-3 fatty acids, vitamins, minerals, etc.), pharmacological interventions (progesterone, corticosteroids, anamoreline, olanzapine, NSAIDs, cannabinoids, melatonin, etc.), and physical exercise [22]. The only available pharmacological intervention is the appetite stimulant megestrol acetate [23], which treats anorexia as one of the causes of cancer-induced cachexia but not the fundamental one. Thus, significant improvement in cancer-induced cachexia symptoms is not seen. Despite the huge effort to develop a novel cancer-induced cachexia treatment, most of these treatments failed phase III clinical trials [24]. Therefore, a search for effective treatment options is still required. Natural products possess unlimited potential due to their beneficial effects on several diseases, not to mention their cost-effectiveness and safety. Chemical drugs for cancer-induced cachexia treatment can cause a burden on the patients, especially when chemotherapy is already negatively affecting them [25]. Thus, we reviewed relevant studies on cancer-induced cachexia, focusing on natural products. Here, we introduce natural products shown to be effective for cancer-induced cachexia and summarize the therapeutic mechanisms.

## 2. Therapeutic Natural Products on Atrophy Induced by Cancer

There is no apparent medication for cancer-induced cachexia due to its multifactorial nature. Since single chemical drugs have failed to show a distinct effect, there are attempts to treat cachectic symptoms by combining several drugs. However, this increased the physical burden on patients [26]. Natural products, composed of several types of bioactive compounds, effectively treat various diseases and symptoms [27]. In addition, natural products have relatively few side effects compared to synthetic drugs. A large portion of natural products are even classified as food, so there is less repulse [28]. These natural products can be consumed steadily, such as tea, without any toxicity. If effective, they may be a new approach for cancer-induced cachexia treatment. In this section, we review studies on natural products which improve cachectic symptoms, and we list therapeutic natural products on muscle atrophy (Table 1) and adipose tissue atrophy (Table 2).

### 2.1. Coix seed Oil (CSO)

CSO, or adlay seed oil, is extracted from *Coix lacryma-jobi* L. var. ma-yuen using standardized pharmaceutical-grade technology. It can be used for hyperlipidemia by reducing blood lipids and exerting antioxidant effects [37]. In addition, CSO is reported to show anticancer effects in preclinical models of sarcoma [38], lung cancer [39], pancreatic cancer [40], liver cancer [41], breast cancer [42], and colon cancer [43]. CSO is shown to be clinically effective [44], and it also helps cancer patients by relieving pain induced by cancer [45]. Liu et al. reported that CSO could ameliorate muscle wasting as well [29]. To induce cancer-induced cachexia, Lewis lung carcinoma cells were injected subcutaneously into C57BL/6 mice. They were then treated with 2.5 mL/kg CSO daily via oral gavage. Administration of CSO significantly reduced body weight loss and prevented the reduction of the gastrocnemius muscle weight and size without changing food intake and tumor size. In this study, CSO inhibited the expression of the full name (MuRF1) in muscle tissue and ameliorated systemic inflammation by decreasing serum IL-6 and tumor necrosis factor-alpha (TNF-α). Systemic inflammation is controlled by nuclear factor kappa B (NF-κB) signaling. In muscle tissue, CSO decreased phosphorylation of p65 (Ser536), an indicator of activated NF-κB signaling. This study suggested that CSO inhibits the NF-kB pathway in muscle tissue by decreasing systemic inflammation. Through this, muscle wasting protein MuRF-1 is reduced, thereby improving muscle wasting. The study also investigated how CSO prevented adipose tissue wasting. In the epididymal adipose tissue, CSO inhibited the phosphorylation of hormone-sensitive lipase (HSL), which induces lipolysis. CSO decreased the phosphorylation of AMP-activated protein kinase (AMPK), an upstream factor of HSL elevated by cancer. However, this result may seem controversial. Rohm et al. reported that the inactivation of AMPK in adipose tissue is correlated with its degradation [21]. Further studies should be conducted to clearly elucidate the action mechanism of CSO and its effect on adipose tissue loss.

### 2.2. Paeonia lactiflora Root

The root of *Paeonia lactiflora* Pall. (RP) has been traditionally used for inflammatory diseases. Due to its immunomodulation effect, RP is used to treat several diseases, including fever, hepatitis, rheumatoid arthritis, dysmenorrhea, and muscle cramping [46]. Studies have demonstrated the traditional effect of RP and have identified new pharmacological effects such as anticancer, anti-diabetes, and neuroprotective effects [47]. Bae et al. reported that RP suppresses muscle wasting induced by lung and colon cancer [30]. The researchers subcutaneously injected Lewis lung carcinoma cells and MC38 colon cancer cells into female C57BL/6 mice. RP treatment suppressed serum TNF-α and IL-6, elevated by cancer allograft. Since elevated serum cytokines induce the activation of NF-κB signaling, reducing systemic inflammation is crucial for ameliorating cachectic symptoms. RP suppressed activation of NF-κB signaling in skeletal muscle tissues. As a result, inflammation is reduced, and thus muscle degradation factors, MuRF1 and MAFbx, were downregulated by RP. On the other hand, food intake was recovered by RP administration compared to the cancer-induced cachexia control group, suggesting the anti-anorexia effect of RP as well. Further research is needed to determine which mechanism is responsible for the anti-cachectic effect of RP, especially by comparing the results from the recovery of appetite and the reduction of NF-κB signaling.

### 2.3. Prunus domestica L.

*Prunus domestica* L., also known as plum, is an edible fruit consumed in many countries. Plums abundantly contain various bioactive components such as polyphenols, chlorogenic acid, rutin, and caffeic acid, and so they are considered “superfoods” that can benefit health [42]. Dried plums show anticancer effects in human hepatocellular carcinoma [43] and anti-inflammatory effects in experimental arthritis [44]. Alsolmei et al. reported that dried plum extract could prevent cancer-induced damage in C2C12 muscle cells [45]. Plum extract increased total protein synthesis by elevating the expression of insulin-like growth factor 1 (IGF-1). Differentiation of C2C12 myoblast was increased, too. In addition, the plum extract reduced muscle cell death induced by conditioned media derived from colon cancer cells. This effect was closely linked to the anti-inflammatory feature of plums. The plum extract reduced TNF-α mediated NF-κB activation. However, this study was only conducted in cell models; thus, further in vivo studies should be carried out to provide evidence for using plums for cancer-induced cachexia treatment.

### 2.4. Peel of Citrus unshiu Markov

*Citrus unshiu* Markov has been traditionally consumed as a food for a long time. The dried peel of *Citrus unshiu* Markov is widely used as an herbal ingredient in East Asia, including Korea, China, and Japan. The *Citrus unshiu* peel (CUP) has been traditionally used to treat digestive dysfunctions and enhance blood circulation. Experimentally, several effects of CUP were proven that possesses anticancer, anti-inflammatory, anti-allergic, anti-bacterial, and anti-diabetic activities. In addition, CUP regulates hyperglycemia as well as lipid metabolism [48,49]. Kim et al. reported that water extract of CUP improved cachectic symptoms in male BALB/c mice bearing CT-26 cells. In this study, CUP improved the reduction of body weight. Tissue weights of gastrocnemius muscle and epididymal adipose tissue were preserved in CUP-treated mice.

They also studied the involved mechanism. In the gastrocnemius muscles, CUP administration decreased MuRF1 and MAFbx by suppressing the NF-κB pathway. Through this, pro-cachectic cytokines such as TNF-α, IL-6, and IL-1β were suppressed. Thereby, muscle degradation was prevented. In the in vitro results, CUP also suppressed cell death and cachectic features in C2C12 myoblasts treated with cancer cell-conditioned media [32]. However, this study did not include an investigation of the mechanism related to adipose tissue loss, although CUP effectively preserved adipose tissue weight as well. Therefore, further study regarding the effect of CUP on adipose tissue seems to be necessary.

### 2.5. Coptidis rhizoma

*Coptidis rhizoma* (CR) is the rhizome of *Ranunculaceae* family, which includes *Coptis chinensis* Franch., *Coptis teeta* Wall., and *Coptis deltoidea* C.Y. Cheng et Hsiao. CR has been traditionally used for diabetes and inflammatory diseases in Asian countries, including Korea, China, Malaysia, Japan, and Singapore [50]. CR is also prescribed to treat pain, fever, and digestive issues [51]. Several types of research have proved that CR has various pharmacological effects on diabetes, atherosclerosis, bacteria, virus, hepatic steatosis, inflammation, and several types of cancers [51]. The reason for the various pharmacological effects seems to be related to more than 120 compounds, such as alkaloids, lignans, phenylpropanoids, phenethyl alcohol, and its glycosides isolated from CR [52]. Iizuka et al. reported that CR showed an anti-cachectic effect. They induced cachexia using human esophageal cancer cell line YES-2 [33] and Colon 26/clone 20 cells [34]. To induce cachexia using colon cancer cells, they subcutaneously injected the cells into male BALB/c mice. CR was administrated from 4 days before injection until the end of the experiment (day 14). A diet supplemented with 1% and 2% of CR were fed to mice. In this experiment, CR significantly prevented weight loss without changes in food intake and tumor size. In addition, weight loss of gastrocnemius muscle and epididymal adipose tissue decreased by CR administration. The anti-cachectic effect of CR seemed to be related to the inflammation response. Serum IL-6 level increased by cancer cells was reduced by CR administration. In tumor, liver, and spleen, IL-6 mRNA and protein expression levels were decreased by CR administration, compared to tumor-bearing mice without treatment. In addition, CR inhibited IL-6 mRNA expression induced by IL-1 in colon 26/clone 20 cells [34]. Iizuka et al. also showed the anti-cachectic possibility of CR in the cachexia model induced by esophageal cancer. For inducing weight loss, YES-2 cells were subcutaneously injected into male BALB/c *nu/nu* mice. They administrated a 1% CR-supplemented diet from 7 days before the injection of the cells until the end of the experiment (day 28). The CR-supplemented diet reduced weight loss without changes in tumor size, food intake, and water intake. In this model, CR significantly suppressed tumor IL-6 levels compared to tumor-bearing mice [33]. Iizuka et al. suggested the potential of CR as a therapeutic agent in cachexia induced by colorectal and esophageal cancer. CR successfully improved body weight and tissue atrophy through the reduction of systemic inflammation. However, the inhibitory mechanism in muscle and adipose tissues was not investigated in both studies. Therefore, further mechanism research is needed for CR to be an anti-cachectic drug.

### 2.6. Arctii fructus

*Arctii fructus* (AF) is a dried fruit of *Arctium lappa*. In herbal medicine, AF has been used to treat and relieve constipation, subdue swelling, and dispel pathogenic wind heat [53]. Recent studies demonstrated that AF improved inflammation, allergy, obesity, cancer, and diabetes [53,54,55,56]. Han et al. reported that AF improved cancer-induced cachexia [35]. They used two types of cancer cachexia models: a mildly induced cachectic mice model and a severely induced cachectic mice model. To induce severe cachexia, they injected CT-26 cells intraperitoneally to mimic diffuse carcinomatosis. After 7 days from CT-26 cell injection, they orally administrated water extract of AF and ethanol extract of AF at a dose of 100 mg/kg once a day until day 16. Although both extracts of AF did not inhibit the weight loss of adipose tissue and muscle, the water extract of AF inhibited body weight loss without food intake change. In addition, water extract of AF decreased mortality. The survival rate of the cachexia group and water extract group was 23.94% and 52.76%, respectively. However, ethanol extract of AF did not show any anti-cachectic effect in the severe cachexia model. In the mild cachectic mice model, both water and ethanol extracts of AF showed an improvement in cachexia. To induce mild cachexia, they subcutaneously injected CT26 cells into male BALB/c mice, and AF was orally administrated from day 7. Administration of water extract of AF and ethanol extract of AF suppressed body weight loss without a change in tumor volume and food intake. In addition, both extracts of AF improved the wasting of adipose tissue and muscle. However, there was no significant effect on cardiac muscle wasting in both severe and mild cachectic models. A549 lung cancer cells are (1 × 10^6^).

### 2.7. Polygonum cuspidatum

*Polygonum cuspidatum* (PC), also known as Japanese knotweed, is a herbaceous perennial plant of the buckwheat family (Polygonaceae). PC contains several active compounds, such as emodin and resveratrol, and is used for treating fever, cough, hepatitis, and pain in traditional medicine [57]. Much research has proved that it has several bioactivities, including anti-bacterial, anti-inflammatory, anti-oxidative, and wound-healing effects [58]. Fang et al. reported that PC improved cancer-induced cachexia [36]. For inducing weight loss, A549 tumor cells were subcutaneously injected into the flank of Balb/c-nude mice (AIN93G). When tumor size reached 100 mm^3^, 2% PC extract in their feed was administrated to the mice for 46 days. PC extract significantly reduced skeletal muscle atrophy and fat browning in A549 tumor-bearing mice. They elucidated that PC extract ameliorated cachexia by inhibiting TCF4/TWIST1 complex-induced parathyroid hormone-related protein (PTHrP) expression, which could maintain bone turnover and skeletal homeostasis [59]. In addition, PC extract inhibited the browning phenotype [60], which could induce cachexia by inducing excessive energy consumption by regulating metabolism-related genes (Pgc1a and Acox1) in epididymal white adipose tissue.

## 3. Therapeutic Natural Products on Atrophy Induced by Cancer Chemotherapeutic Agents

Chemotherapy uses a chemical drug to kill fast-growing cells, such as several cancer cells [61]. Although cancer therapy with the chemical drug is a mainstream treatment for cancer, several studies have reported that some chemotherapeutic drugs, including doxorubicin and cisplatin, can induce the development and progression of cachexia [62,63,64]. Doxorubicin is an anthracycline cytostatic agent used for treating solid tumors. Doxorubicin-induced cell death by interfering with DNA replication and transcription. However, healthy cells are also affected due to the non-specific mechanism of action [65]. Hiensch et al. reported that doxorubicin decreased skeletal muscle in preclinical models [64]. Cisplatin is a chemotherapeutic drug for treating several types of cancers found in the lung, ovary, testes, and bladder. In addition, it can be used for metastatic cancer as the first standard treatment [66]. However, it has been reported that cisplatin induces several side effects, such as acute kidney injury and renal tubular necrosis. In addition, cisplatin decreased weight induced by muscle atrophy. Patients with cachectic symptoms are in an extremely grievous situation due to decreased response to cancer therapy [66,67]. According to the study reported by Daumrauer et al., cisplatin promoted muscle atrophy by increasing MuRF-1 via NF-κB activation [68]. Worst of all, the cancer drug combination for stage IV patients induced muscle wasting. It has been reported that folfiri, a mixture of 5-fluorouracil, leucovorin, and irinotecan for stage IV colon cancer, induced cachexia by activating ERK1/2 and p38 [25]. According to a study by Pin et al., tumor-transplanted mice with folfiri treatment lost more weight than tumor-transplanted mice without the drug [63]. Therefore, cachexia caused by cancer chemotherapeutic drugs is not only difficult to improve, but there is also no clear treatment. In this part, we introduced several natural products that can improve the cachexia induced by chemotherapy, and they are listed in Table 3.

### 3.1. Scutellaria baicalensis Georgi

*Scutellaria baicalensis* Georgi has been widely used as herbal medicine for treating various diseases, such as respiratory infections, diarrhea, dysentery, and hypertension [73,74]. Huang et al. have reported *Scutellaria baicalensis* (SB) ameliorates cachexia induced by cisplatin [69]. To mimic the cachexia model induced by cisplatin, Lewis lung carcinoma cells were subcutaneously injected into male C57BL/6J mice. Paradoxically, the treatment of cisplatin decreased body weight by inducing muscle weight loss. However, the administration of SB significantly reduced weight loss. The atrophy of muscle tissue induced by cisplatin was improved by the administration of SB, and had no effect on adipose tissue. In this study, the administration of SB enhanced the therapeutic ability of cisplatin. Although SB did not reduce tumor size, simultaneous administration of cisplatin and SB reduced the tumor size more than cisplatin without the SB group. Through this experiment, it was confirmed that the decreased therapeutic effect of the anticancer drug, which is one of the symptoms of cachexia, was improved by SB administration. In this study, SB suppressed cisplatin, mediating weight loss by improving muscle wasting syndrome. This anti-cachectic effect of SB improved the anticancer effect of cisplatin, and, consequently, cisplatin was able to reduce the tumor size more effectively [69].

### 3.2. Citrus unshiu Peel

The anti-cachectic effect of *Citrus unshiu* peel was first reported in 2016, and it can improve cancer-induced cachexia [32]; thus, *Citrus unshiu* peel already showed a potential therapeutic effect. In addition, Tahaghoghi-Hajghorbani et al. reported that it is also effective in cachexia induced by doxorubicin, an anticancer drug [70]. In this study, they used two-time points: prophylactic treatment, which was treated for 10 days before injecting cancer cells, and therapeutic treatment, which was treated 14 days after injecting cancer cells. 6 mg/kg of doxorubicin was treated twice a week with or without the extract of *Citrus unshiu* peel 14 days after the cancer cell injection. Although weight loss was observed in all cancer cell injected groups, the extract of *Citrus unshiu* peel group experienced significantly improved weight loss compared to the doxorubicin administered group at 20 days after transplantation. However, there was no significant difference in the effect on day 24. In addition, a combined administration of the extract of *Citrus unshiu* peel and doxorubicin did not show a significant difference compared to doxorubicin administration alone. Interestingly, prophylactic groups administered with the extract 10 days earlier, before cancer cell injection, did not improve weight loss, regardless of whether the extract of *Citrus unshiu* peel was used with or without doxorubicin. In this study, weight loss improved by decreasing IL6, Il-1β, TNF-α, and Malondialdehyde–Thiobarbituric acid (MDA) levels in serum. However, at the end of the experiment, the tumor size of all groups was decreased compared to the non-treated cancer cell injected group. Therefore, it is not clear from this study whether the weight improvement was due to the treatment of cancer or improving cachexia.

### 3.3. Panax ginseng

The root of *Panax ginseng* Meyer has been used for treating several diseases, such as neurological, cardiovascular, and autoimmune diseases, by enhancing immunity [75,76]. It has been reported that the extract of *Panax ginseng* has a preventive effect against various human cancers, including esophageal cancer, stomach cancer, colorectal cancer, liver cancer, pancreatic cancer, laryngeal cancer, lung cancer, and ovarian cancer [77]. Lobina et al. have reported that the extract of *Panax ginseng* can protect against weight loss induced by the cancer drug cisplatin [71]. For inducing weight loss, cisplatin was administered to normal rats twice a week for a total of 5 weeks; 1 mg/kg (first injection) and 2 mg/kg (second injection). For the same period, 25 and 50 mg/kg of the extract was administered orally every day. The signs of malaise, based on muscle flaccidity, hindlimb weakness, paw paleness, piloerection, gastrointestinal disorders, and tremors/convulsions, induced by cisplatin were improved by the administration of the extract of *Panax ginseng*. Administration of the *Panax ginseng* extract improved the weight loss caused by cisplatin compared to the cisplatin-injected group. On the treadmill, which can test the strength of skeletal muscle, administration of *Panax ginseng* extract increased motor performance compared to the cisplatin injected group. In addition, hypothermia induced by cisplatin injection was prevented by *Panax ginseng* extract. This study indicated that *Panax ginseng* extracts protected cachexia in a rat model. Although they suggested that *Panax ginseng* extract could be a therapeutic drug for supporting oncology care, there was no specific mechanism for the anti-cachectic effect of ginseng extract.

### 3.4. Magnoliae cortex

*Magnoliae cortex* (MC) is the bark of the stems and roots of *Magnolia officinalis* Rehd. et Wils; it is used for acute diarrhea, regurgitation, vomiting, and cramping abdominal pain [78]. It has been reported that MC contains honokiol and magnolol as the main bioactive compounds. They are phenolic compounds and suppress the inflammatory response by decreasing the expression of TNF-α, IL-6, nitric oxide (NO), and prostaglandin E2 (PGE2) [79,80,81]. Hong et al. [72] reported that MC ameliorated chemotherapy-induced muscle atrophy by regulating M2 macrophage polarization. To induce muscle wasting, 2.5 mg/kg of cisplatin was intraperitoneally injected into C57BL/6 mice with oral administration of MC extract (50, 100, and 200 mg/kg) every 3 days for 12 times. Administration of MC (100 and 200 mg/kg) significantly decreased cisplatin-induced body weight loss, skeletal muscle loss, and muscle strength weakness. They found that administration of MC increased the expression of M2 macrophage markers and decreased the expression of M1-specific markers without interference with the cisplatin anticancer effect in colon cancer.

## 4. The Role of AMPK in Cancer-Induced Cachexia and Related Natural Products

Metabolic regulation plays an important role in cancer-induced cachexia, characterized by significant weight loss, muscle wasting, and weakness [82]. Cancer-induced cachexia is a multifactorial syndrome that is thought to result from a combination of metabolic as well as inflammation and neural factors. In cancer-induced cachexia, metabolic regulation is disrupted, leading to alterations in the way the body processes energy and nutrients [5]. Understanding the metabolic changes that occur in cancer-induced cachexia is important for the development of effective treatments.

AMPK is a metabolic regulator that maintains energy homeostasis in cells. In cancer-induced cachexia, it has been reported that AMPK could play a key role in regulating energy production and consumption, and it has been suggested to contribute to the development of muscle wasting and weight loss seen in cachexia [21,83,84]. In cancer-induced cachexia, AMPK activation may contribute to muscle wasting by promoting protein breakdown and inhibiting protein synthesis. Liu et al. reported that *Coix seed* oil suppressed lipolysis of adipose tissues by inhibiting the AMPK activated by cachexia [29]. However, Han et al. showed the opposite result [35]. They treated the extract of *Arctii fructus*, and AMPK phosphorylation suppressed by cancer-induced cachexia restored in adipose tissues. Rohm et al. also support the result that the inactivation of AMPK represents a critical pathophysiological event in adipose tissue dysfunction during cancer-induced cachexia [21]. However, the exact role of AMPK in cancer-induced cachexia is still controversial, and it is possible that AMPK may play a complex and multifaceted role in the development of cachexia. Oliveira et al. reported that metformin, which is a well-known AMPK activator to treat type 2 diabetes used for more than 60 years, could regulate muscle protein metabolism in cancer-induced cachexia [85]. However, Hall et al. reported that activation of AMPK was associated with muscle wasting in only a syndrome of inflammatory-driven muscle atrophy. They elucidated that AMPK agonist AICAR, not metformin, could suppress IFNγ/TNFα-induced cachexia symptoms [83]. Therefore, further research is needed to fully understand the mechanisms by which AMPK contributes to cachexia.

## 5. Conclusions and Future Directions

In this review paper, we have summarized research papers on the anti-cachectic effect regarding the single extraction of natural products. Although multi-herbal decoctions are frequently used in traditional medicine in Asian countries, there is research on herbal medicine prescriptions. In addition, various synthetic drugs are used to ameliorate wasting syndrome and anticancer drugs [5]; thus, caution should be exercised in using supplemental drugs other than clinically validated drugs. Therefore, we tried to help cachexia treatment through this review, mainly with a single extract, to minimize the unexpected effects on cachexia patients when trying natural products to ameliorate cachectic symptoms. Some natural substances listed in this review could also be used for food, so we expect them to be used as herbal tea during cachexia treatment. While it is crucial for the quality of life (QOL) of patients, little is known about treatments for cancer cachexia; thus, various studies should be attempted. Several types of synthetic drugs, including progesterone analogs, corticosteroids, megestrol acetate, anamorelin, cannabinoids, and NSAIDS are attempts to ameliorate symptoms. However, the US Food and Drug Administration (FDA) did not approve them for treatment of cancer cachexia due to the side effects and the fact that they had no significant effect in clinical trials [22]. Although several studies reported that combining more than one drug is useful, there was no significant difference with a single drug in clinical trials [22,86]. The therapeutic effect is also not strong. Many researchers tried various methods, such as alternative medicine, to improve cachexia [87]. However, it is important to be careful about taking medicinal products, such as natural products and herbal medicines, when synthetic drugs are prescribed [88,89,90]. Although several multi-component extracts of herbal medicine are reported to be effective in improving cachexia, they are only used in East Asia [91]. In addition, there are difficulties in using them as an adjuvant or treatment for cachexia because of the lack of clinical studies on the interaction mechanism between herbal medicine and cancer drugs [92]. Therefore, we summarized the effect of a single natural extract on cachexia to help patients when they consider the secondary options for ameliorating cachectic symptoms.

Historically, natural products have served an important role in drug discovery in various therapeutic areas, such as cancer and infectious diseases [93]. Systemic inflammation is considered an important therapeutic target pathway for cachexia [94]. Most natural products listed in this review paper improved cachexia symptoms by suppressing systemic inflammation. For this reason, the administration of natural products for improving cachexia is reasonable. In addition, natural products are generally better than synthetic drugs in improving their side effects [28,95]. In cancer, docetaxel, 5-FU, and cisplatin are widely used as anticancer drugs [96]. Although those drugs improved cancer by reducing the size of the tumor in mice, cachectic phenotypes such as weight loss and atrophy of muscle/fat were induced by cancer drugs. It seems that the systemic inflammatory response was induced by the administration of the anticancer drug by increasing inflammatory factors, including TNF-alpha and IL-6, which could deteriorate the cachexia [97,98,99]. Therefore, suppressing the size of the tumor cannot be the answer to improving cachexia. In some studies, weight loss and reduction in tumor size were simultaneously observed [69,70]. Since mild cancers do not induce weight loss or cachexia, it is difficult to form a conclusion about whether cachexia is ameliorated by drugs or whether cachexia is not induced due to mild cancer. In cachexia studies, therefore, the drug effect on anti-cachexia should be confirmed after inducing weight loss, not simultaneous cancer cell injection and drug administration. Since cancer-induced cachexia reduces the amount of muscle and adipose tissue in the body despite sufficient nutrient supply, it is important to suppress the reduction of muscle and adipose tissue by administering drugs to improve cachexia [10,11]. Therefore, tracking the weight change of muscle and adipose tissue is very important to determine drug efficacy.

It is known that not only the weight of muscle but also the weight of fat is closely related to determining the mortality in cancer-induced cachexia [100]. So far, research on the treatment mechanism of cachexia is not sufficient; therefore, it is important to elucidate how the administration of natural products prevents the atrophy of each tissue in cancer conditions. However, several studies were only focused on muscle atrophy [30,31]. In addition to the aforementioned paper, weight loss improvement was reported in several papers, but the specific mechanism and phenomenon were not observed in the paper, and only weight changes were reported [101]. In Chen and Wang’s study [102], the weight loss inhibitory effect of *Brucea javanica* extract was demonstrated, but the weight for each tissue was not presented in the paper. Moreover, the weight improvement and cancer size reduction appeared to be similar. Therefore, more detailed studies on the effect of improving cachexia are required.

In modern medicine, cancer can be treated with several methods, such as surgery, radiation therapy, chemotherapy, or combined methods. However, each method has been shown to be high risk, with widespread damage from surgery, radiation, and chemotherapeutic drugs [103]. Since conventional therapy could be successful to some extent in regards to life span, even though the main drawbacks are its poor bioavailability and adverse side effects, it is difficult to avoid using synthetic drugs during cancer treatment for alternative treatment [103,104]. Therefore, more in depth research on the combined administration of anticancer drugs with herbal medicine is required. Because natural products contain bioactive compounds, combined administration has to be more careful. This is also the reason why our thesis focused on a single natural product, not an extract of multiple natural product mixtures.

In herbal medicine research, cachexia is also studied in traditional medicine, consisting of various natural product mixtures (Yukgunja, Sipjeondaebo), and their efficacy has been reported. Some prescriptions are in clinical trials [105,106,107,108,109,110,111,112]. Kang et al. provided scientific evidence on the anti-cachectic effect of the natural product mixture ‘Yukgunja’ in a clinical trial. Although their study was the first clinical trial in Korea, the number of patients was relatively small (fewer than 100 subjects) and only focused on anorexia [105,113]. Cheon et al. also performed a clinical trial using the natural product mixture ‘Sipjeondaebo’ only on anorexia [107]. There has been little research on clinical trials of herbal medicine prescriptions with detailed molecular mechanisms in muscle and adipose tissues, so there are many limitations in the actual clinical use of the natural product. Therefore, it is reasonable to study a single extract of a natural product preferentially rather than such a complex prescription to patients using anticancer drugs to reduce this risk. In future studies, it is necessary to increase the possibility of using natural products in cachexia by studying the side effects and expected effects of combined administration with anticancer drugs.

Although more research is needed to develop drugs for cancer-induced cachexia, natural products could contribute to ameliorating cancer-induced cachexia for elevating the quality of life in cancer patients. Therefore, further investigations are expected to find a novel natural product and explore the role of AMPK that improves understanding of cancer-induced cachexia through mechanistic studies.

## Figures and Tables

**Table 1:** Natural products that improve muscle atrophy.

Natural Product	Cachexia Model	Administration	Mechanism	Reference
*Coix seed* oil	C57BL/6 mice were injected subcutaneously with Lewis lung carcinoma cells for 24 days.	Cachectic mice were treated with 2.5 mL/kg *Coix seed* oil daily gavage.	Down-regulation of MuRF-1 via NF-κB signaling.	Liu, H. 2019 [29]
Root of *Paeonia lactiflora* Pall.	C57BL/6 mice were injected subcutaneously with Lewis lung carcinoma cells and MC38 cells for 4 weeks.	During the last 2 weeks of the experiment, 30% ethanol extract of the root of *Paeonia lactiflora* (50, 100, and 200 mg/kg/day) was orally treated.	1. Food intake recover 2. Downregulation of MuRF-1 and MAFbx via NF-κB signaling.	Bae, T. 2020 [30]
*Prunus domestica* L.	To mimic cachexia condition, C2C12 are maintained in colon cancer cell cultured media	Treatment to C2C12 myocytes	1. Protein synthesis through IGF-1 2. Decreasing NF-κB signaling by suppressing TNF-α.	Alsolmei, F.A. 2019 [31]
*Citrus unshiu* Peel Extract	Male BALB/c mice were subcutaneously injected with CT-26 cells for 27 days.	From day 10, after the tumor cell injection, CUP was orally treated (250 and 500 mg/kg/day).	1. Decreasing NF-κB signaling by suppressing cytokines such as TNF-α, IL-6, and IL-1β.	Kim et al., 2016 [32]
*Coptidis rhizoma*	Male BALB/c *nu/nu* mice injected subcutaneously with colon 26/clone 20 cells.	From 4 days before injection until the end of the experiment (day 14).	Decreasing IL-6 levels.	Iizuka, N. 2000 [33]
*Coptidis rhizoma*	Male BALB/c mice injected subcutaneously with YES-2 cells.	From 7 days before injection until the end of the experiment (day 28).	Decreasing IL-6 levels.	Iizuka, N. 2002 [34]
*Arctii fructus*	Male BALB/c mice injected subcutaneously (mild model) and intraperitoneally (severe model) with CT-26 cells.	Mild model: day 7~16 Severe model: day 9~27 100 mg/kg/day.	Decreasing IL-6 levels Murf1, MAfbx.	Han et al., 2020 [35]
*Polygonum cuspidatum*	A549 tumor-bearing BALB/c-nu mice were subcutaneously injected into the flank.	2% PC extract in their feed for 46 days.	Decreasing muscle atrophy-related genes (mstn, fbxo32, trim 63).	Fang et al., 2022 [36]

**Table 2:** Natural products that improve adipose tissue atrophy.

Natural Product	Cachexia Model	Administration	Mechanism	Reference
*Coix seed* oil	C57BL/6 mice were injected subcutaneously with Lewis lung carcinoma cells for 24 day.	Cachectic mice were treated with daily gavage of 2.5 mL/kg *Coix seed* oil (CA + Coix group).	Inhibition of cancer-induced lipolysis by deactivating HSL and AMPK.	Liu, H. 2019 [29]
*Citrus unshiu* Peel Extract	Male BALB/c mice have injected subcutaneously with CT-26 cells for 27 days.	From day 10, after the tumor cell injection, CUP was orally treated (250, 500 mg/kg/day).	1. Decreasing NF-κB signaling by suppressing cytokines such as TNF-α, IL-6, and IL-1β.	Kim et al., 2016 [32]
*Coptidis rhizoma*	Male BALB/c mice injected subcutaneously with YES-2 cells.	From 7 days before injection until the end of the experiment (day 28).	Decreasing IL-6 levels.	Iizuka, N. 2000 [33]
*Arctii fructus*	Male BALB/c mice injected subcutaneously (mild model) and intraperitoneally (severe model) with CT-26 cells.	Mild model: day 7~16 Severe model: day 9~27 100 mg/kg/day.	Decreasing IL-6 levels. Decreasing UCP1 by restoring AMPK activation.	Han et al., 2020 [35]
*Polygonum cuspidatum*	A549 tumor-bearing BALB/c-nu mice were subcutaneously injected into the flank.	2% PC extract in their feed for 46 days.	Decreasing metabolism in epidydimal adipose tissue. (PGC1 alpha, Acox1, Glut1).	Fang et al., 2022 [36]

**Table 3:** Natural products that ameliorate atrophy induced by cancer chemotherapeutic agents.

Natural Product	Cachexia Model	Administration	Mechanism	Reference
*Scutellaria baicalensis* Georgi	Male BALB/c mice injected subcutaneously with Lewis lung carcinoma cells for 23 days. From day 7 to day 21, 3 mg/kg of cisplatin was intraperitoneally injected every 2 days.	From day 7 to day 23, 300 mg/kg of SB was orally administered daily.	Ameliorating weight loss by inhibiting muscle atrophy.	Huang et al., 2019 [69]
*Citrus unshiu* peels	BALB/c male mice aged 6–8 weeks. 5 × 10^5^ C26 cells in 0.2 mL RPMI were injected subcutaneously at the flank.	Prophylactic treatment: 10 days before C26 cell injection. Therapeutic effect: From 14 days after C26 cell injection to the end of the experiment. The extract of *Citrus unshiu* peels (350 mg/kg) was orally administrated every day, and 6 mg/kg of Dox was intraperitoneally administrated twice a week.	Decreasing serum levels of IL6, Il-1β, TNF-α, and Malondialdehyde–Thiobarbituric acid (MDA).	Tahaghoghi-Hajghorbani et al., 2019 [70]
*Panax ginseng*	Male Wistar rats (average bodyweight approximately 180 g), 1–2 mg/kg of cisplatin was intraperitoneally administered twice weekly for 5 consecutive weeks.	Panax ginseng extracts (25 and 50 mg/kg) were treated daily by intragastric administration.		Lobina et al., 2014 [71]
*Magnoliae cortex*	2.5 mg/kg of cisplatin was intraperitoneally injected into C57BL/6 mice every 3 days 12 times.	MC extract (50, 100, and 200 mg/kg) was orally administrated with cisplatin injection.	Regulation of M2 macrophage polarization in skeletal muscles: M2 macrophage markers increase (MRC1, CD163, TGF-β, and Arg-1). M1-specific markers decreased (NOS2, TNF-α, in skeletal muscle.	Hong et al., 2021 [72]

## Data Availability

Not applicable.
